# Single-cell analysis identifies BASP1 as a driver of drug resistance and cell plasticity in oral squamous cell carcinoma

**DOI:** 10.1016/j.jbc.2025.111126

**Published:** 2026-01-06

**Authors:** Abinash Behera, Sudeshna Datta, Sibasish Mohanty, Pallavi Mohapatra, Shamima Azma Ansari, Sreeparna Podder, Rachna Rath, Dillip Kumar Muduly, Rajeeb K. Swain, Sunil K. Raghav, Rupesh Dash

**Affiliations:** 1 Institute of Life Sciences, Bhubaneswar, Odisha, India; 2 Regional Centre for Biotechnology, Faridabad, India; 3 School of Biotechnology, KIIT University, Bhubaneswar, Odisha, India; 4 Department of Oral and Maxillofacial Pathology, SCB Dental College and Hospital, Cuttack, Odisha, India; 5 Department of Surgical Oncology, All India Institute of Medical Sciences, Bhubaneswar, Odisha, India

**Keywords:** chemoresistance, cisplatin, OSCC, EMT, head and neck squamous cell carcinoma (HNSCC), single-cell RNA sequencing

## Abstract

Despite initial positive responses with chemotherapy, many cancer patients experience relapse, continued tumor growth, and metastatic spread due to drug resistance. It is well documented that a rare population of phenotypically heterogeneous cells contributes to intratumour heterogeneity and drug resistance. To date, these rare populations are poorly characterized. To identify the potential role of these rare populations in drug resistance, here we have performed single-cell RNA sequencing of human oral squamous cell carcinomas lines presenting with sensitive, early, and late cisplatin-resistance patterns. The single-cell RNA-sequencing data identified two different transitional clusters within the three, sensitive, early, and late cisplatin-resistant major clusters. The differential gene expression profile and deregulated pathways analysis suggested Brain Abundant Membrane-Attached Signal Protein 1 (BASP1) as a major upregulated gene not only in major drug-resistant clusters but also in transitional clusters. Selective knockdown of BASP1 reverses epithelial to mesenchymal transition (EMT) phenotype in cisplatin-resistant cells and restores cisplatin-induced cell death. Mechanistically, BASP1 positively regulates LIN7A expression through phosphorylation of RAC-alpha serine/threonine-protein kinase as well as by supressing microRNA hsa-mir-501-3p, which in turn induces β-catenin-mediated EMT in chemoresistant cells. Overall, our study demonstrates that BASP1 acts as a key regulator of EMT in cisplatin-resistant oral squamous cell carcinoma and represents a promising therapeutic target to overcome drug resistance in advanced stages of the disease.

Oral squamous cell carcinomas (OSCC) that originate from mucosal epithelium are the most prevalent neoplasm among men in India, with approximately 80,000 new cases being diagnosed every year (1). Unfortunately, almost 70% of the patients are present with advanced disease. In a 2025 multi-centre clinical study of 200 oral cancer patients in India, a marked male predominance was observed (86.5%, n = 173) over female patients (13.5%, n = 27). Among them 80.0% (n = 160) of the total cases use of some form of tobacco, *i.e.,* “betel quid” and “gutkha, strongly suggesting that tobacco consumption is the central driver for the gender disparity in oral cancer incidence in Indian population. Many patients seek medical care only at advanced stages because of low awareness and inadequate early screening (2). The high prevalence combined with late diagnosis and other socio-cultural factors, explains the disproportionately higher burden of OSCC in Indian men. The treatment regimen of OSCC involves surgical removal of tumor followed by concomitant chemoradiotherapy (3). Cisplatin alone or in combination with 5FU and Docetaxel (TPF) is the most common chemotherapy regimen for advanced OSCC. Despite initial positive response, many patients experience relapse, continued tumor growth, and metastatic spread due to drug resistance. Chemoresistance is one of the major causes of treatment failure in OSCC. The chemotherapy drugs competently induce cell death in the rapidly dividing cancer cells, but poorly eliminate a small or rare population of cells that are slow-growing and escape the cytotoxic effect of the drug (4). It is hypothesized that this rare population of cells contributes to drug resistance, recurrence, and metastatic spread.

Tumor mass exhibits a great extent of heterogeneity in genetic alteration, cell morphology, proliferative capacity, and response to chemotherapeutic drugs (5). The heterogeneity in tumor cells contributes to a rare population of cells that are resistant to the cytotoxic effect of chemotherapy drugs. In literature, these rare populations are attributed as cancer stem cells or quiescent cells or having an epithelial to mesenchymal transition (EMT) phenotype. It is well conceived that cancer stem cells (CSC) are a small population of heterogeneous tumors that are not only resistant to chemotherapy but also have enhanced tumor-initiating abilities, which contribute to drug resistance and recurrence (6). Despite investigations with numerous cutting-edge technologies to characterize CSCs, they pose a significant clinical challenge in overcoming cancer chemoresistance (7). Unfortunately, the chemotherapy drugs enrich the drug-resistant clones of CSCs to acquire drug resistance, followed by continued tumor growth (8). Similarly, quiescent cells are mostly arrested in the cell cycle phase G0 as they do not undergo frequent cell division. The quiescent state is generally induced when cells are deprived of nutrients or oxygen, which has the hallmark of increased expression of cell cycle suppressor p27 and decreased expression of proliferative signal ki67 (9). A rare population of cancer cells induces a quiescent state to escape the cytotoxic effect of chemotherapy. It is noteworthy to mention that chemotherapeutic drugs target cancer cells that are in the proliferative stage, *i.e.*, S/G2/M phase of the cell cycle (10). In addition to this, it is also reported that in tumor microenvironment, the quiescent cells elevate hypoxia-induced signals that exhaust cytotoxic T cells, and that’s how quiescent cells evade immune surveillance (11). Upon sensing the favorable condition with reduced chemotherapeutic activity, these quiescent cells can resume cell cycle, leading to continued tumor growth and drug resistance (12). Unlike quiescent cells, CSCs exist in any phase of cell cycle; however, both CSCs and quiescent cells get enriched with chemotherapy treatment and lead to drug resistance. Similarly, few literatures suggest that heterogeneous subpopulation of cells that exists in the tumor mass shows chemotherapy-induced EMT phenotype. The EMT not only contributes to changes in morphology and motility of cancer cells to augment invasion, but it also contributes to other aspects of tumorigenesis, like drug resistance (13). Again, the chemotherapy drugs select clones of cells that have enhanced EMT transcription factors like ZEB1, SNAIL, and VIM (14). These subpopulations of cells have elevated efflux pumps like ABC transporters, and hence they escape the cell death induced by chemotherapy (15). In addition to this, cells having EMT hallmarks also gain a CSC phenotype that contributes to drug resistance (16). Categorization of these drug-resistant phenotypes is the outcome of a sporadic study where these cells were isolated using cell sorter, and their role in drug resistance is being investigated. Hence, it is important to identify and explore the role of these subpopulations of cells in drug resistance in isogenic cancer cells.

In this study, we hypothesize that there is existence of subpopulation of drug-resistant cells among isogenic cancer cells, and these subpopulations of cells differ in terms of their global gene expression profile. The gene expression profile of these subpopulations can help us to understand the molecular mechanism behind cisplatin resistance in OSCC. We also predict that, due to increased resolution and the ability to sense the clonal evolution; these rare populations of cells can be identified and characterized through single-cell RNA sequencing. Hence, we performed single-cell RNA sequencing of human OSCC lines presenting with sensitive, early, and late cisplatin-resistance patterns to identify Brain Abundant Membrane-Attached Signal Protein 1 (BASP1) as a major factor for EMT and cisplatin resistance in OSCC. BASP1 is a 22 kDa acidic protein, a neuronal growth-associated protein found to be enriched in neuronal tissue in the brain (17). BASP1 interacts with cholesterol to regulate chromatin remodelling and the direction of transcription programs that control cell differentiation (18). Other than neuronal tissues, BASP1 expression is found to be upregulated in a spectrum of cancers, including lung and cervical cancers (19, 20).

## Results

### Single-cell RNA sequencing revealed BASP1 as a sustainer of cisplatin resistance

**Figure 1:**
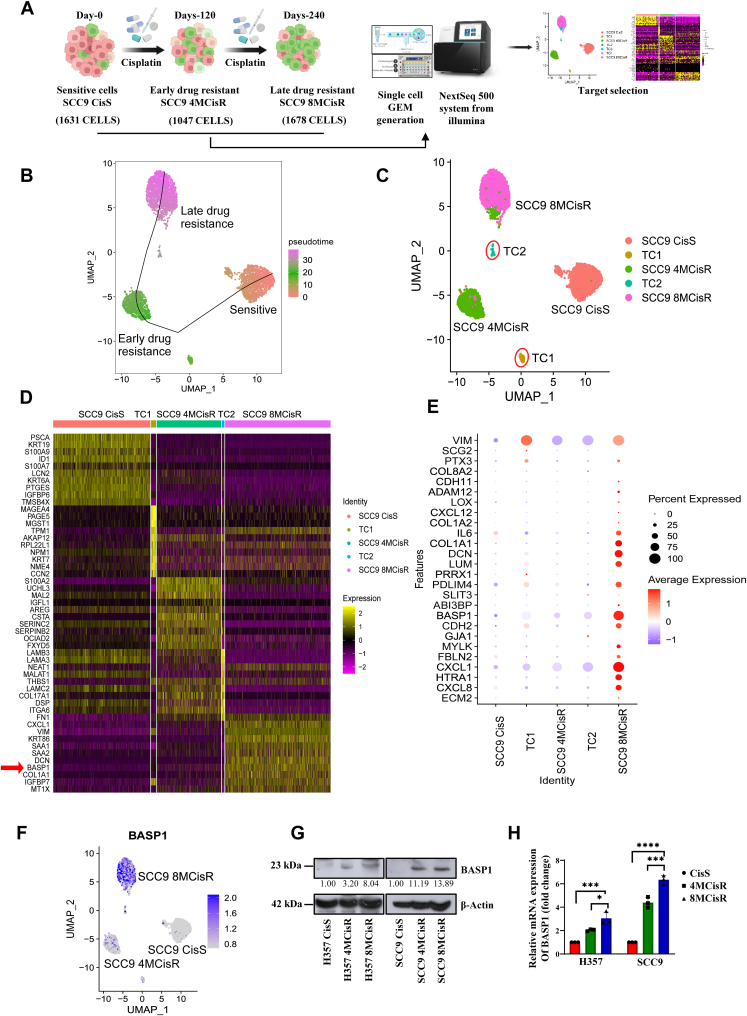
**Single-cell RNA-seq identifies BASP1 expression is elevated in chemoresistant OSCC.***A,* schematic representation of the experimental workflow for scRNA-seq analysis, of sensitive, early, and late cisplatin-resistant OSCC cells. *B,* uniform manifold approximation and projection (UMAP) visualization of scRNA-seq data of human OSCC lines (SCC9) presenting with sensitive (SCC9 CisS), early (SCC9 4MCisR) and late cisplatin-resistance (SCC9 8MCisR) patterns. The line shows slingshot pseudotime analysis represents the trajectory of cells transitioning from sensitive to a resistant pattern. *C,* UMAP projection of scRNA-seq data, highlighting three major cell clusters of (SCC9 CisS), early (SCC9 4MCisR), late (SCC9 8MCisR), and rare transitional clusters (TC) depicted as TC1, TC2. Cells are clustered based on their gene expression profiles and color-coded to distinguish each of them. *D,* heatmap of top 10 differentially upregulated genes in each identified cell cluster and transitional cluster (TC1 and TC2). *E*, dot plot showing the average scaled expression of few top differentially regulated genes for annotated cell types and the percentage of cells expressing each gene in major clusters and transitional cluster as depicted in *panel D*. *F,* UMAP plot depicting the expression of the BASP1 gene in three major cluster and transitional clusters. The intensity of blue color represents the level of gene expression in individual cells. *G,* cell lysates from indicated resistant and sensitive OSCC cell lines were subjected to immunoblotting (n = 3) using antibodies against BASP1 and β-Actin. *H,* relative mRNA expression of BASP1 was analyzed by quantitative real-time PCR (qRT-PCR) in the indicated cells. Data are presented as mean ± SEM (n = 3), ∗*p* ≤ 0.05 by 1-way ANOVA. OSCC, oral squamous cell carcinomas.

To understand the potential role of the rare subpopulation of cells in acquired drug resistance, we performed scRNA-seq of OSCC lines presenting with sensitive, early, and late cisplatin-resistance patterns (Fig. 1*A*). Earlier, we established early and late resistant lines by treating cisplatin to OSCC lines SCC9 for 4 months (early resistance) and 8 months (late resistance), respectively. The parental sensitive SCC9 line is termed as SCC9 CisS, the early cisplatin-resistant cells as SCC9 CisR 4M, and the late resistant cells as SCC9 CisR 8M (21). Through scRNA-seq gene expression analysis and uniform manifold approximation and projection, we found three major clusters, *i.e.*, sensitive, early, and late drug resistance, with cell counts of 1541, 1021, and 1673, respectively (Fig. S1, *A*–*D*). As expected, the pseudo-time analysis showed cell pattern trajectory from sensitive to early resistance and then late resistance clusters (Fig. 1*B*). However, we found two transitional rare clusters and named them to be TC1 and TC2 (Fig. 1*C*). The TC1 was found between sensitive and early resistant cluster and TC2 between early and late resistant cluster (Figs. 1*C* and S1*A*). We found the origin of TC1 from sensitive cluster, TC2 from early resistant cluster (Fig. S1, *A*–*D*). Next, we performed differential gene expression analysis and explored the genes that are not only upregulated in the resistant cluster but also in the transitional cluster. Our analysis showed a progressive upregulation of BASP1 expression from the early to the late resistant clusters, with elevated levels also observed in the transitional clusters TC1 and TC2 (Fig. 1, *D*–*F*). To validate our findings from scRNA-seq, we performed immunoblotting and qRT-PCR against BASP1 in SCC9 as well as H357 cisplatin sensitive, early and late resistant cells, where we found that BASP1 is upregulated in early and late resistant OSCC lines as compared to the sensitive counterpart (Fig. 1, *G* and *H*).

### BASP1 is an important target to overcome cisplatin resistance in OSCC

**Figure 2:**
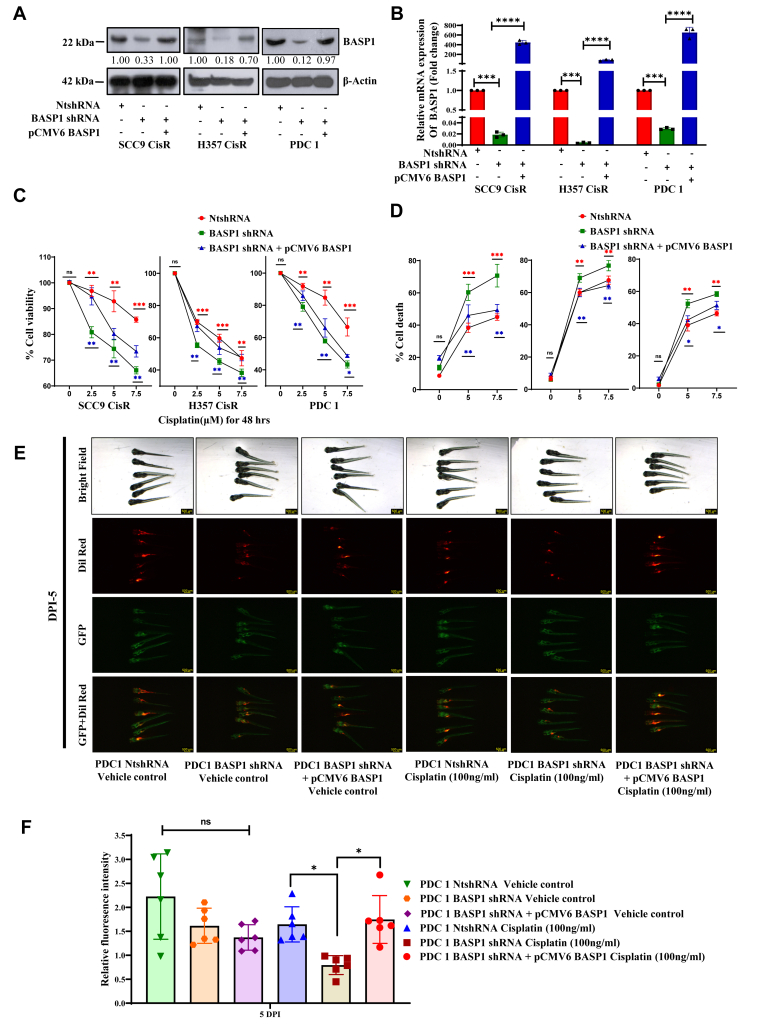
**Ectopic BASP1 overexpression rescued the drug-resistant phenotype in BASP1 KD cells.***A,* cisplatin-resistant OSCC cell lines were stably transfected with NtshRNA and BASP1 shRNA (targeting the 3′UTR of BASP1 mRNA using lentiviral approach, and for ectopic overexpression, BASP1 KD cells were transiently transfected with pCMV6 BASP1 (MYC-(DYKDDDDK) DDK tagged), as described in the methods section. Lysates were collected from the indicated cells, and immunoblotting (n = 3) was performed using anti-BASP1 and β-Actin antibodies. *B,* relative mRNA expression of BASP1 was analyzed by quantitative real-time PCR (qRT-PCR) in the indicated cells. Data are presented as mean ± SEM (n = 3), ∗∗∗*p* ≤ 0.0001, ∗∗∗∗*p* ≤ 0.0001 by 1-way ANOVA. *C,* BASP1 was overexpressed in chemoresistant cells stably expressing BASP1 shRNA and treated with cisplatin at indicated concentrations for 48h. Cell viability was then determined by MTT assay, mean ± SEM (n = 3), ∗*p* ≤ 0.05 by 2-way ANOVA. *D,* Indicated cells were treated with cisplatin at indicated concentration for 48h, and cell death was determined by Annexin V/7-AAD staining and flow cytometry. Dot plot shows the percentage of cell death in each treatment group, mean ± SEM (n = 3), ∗*p* ≤ 0.05 by 2-way ANOVA. *E,* lateral view of fluorescent transgenic Tg(etsrp:EGFP) zebrafish embryos at Day 0 and Day 5 post-injection with Dil-Red-stained PDC1 NtshRNA control, BASP1 shRNA and pCMV6 BASP1 over expressed cells. Embryos were treated with either cisplatin or vehicle control. Tumor growth was assessed by comparing fluorescence intensity at Day 5 to Day 0. (n = 6). *F,* graphs indicating relative fluorescence intensity which was quantified using ImageJ software from *panel E*. (∗*p* ≤ 0.05 by 2-way ANOVA). KD, knockdown cell; BASP1, Brain Abundant Membrane-Attached Signal Protein 1.

To explore if BASP1 can be a potential target to overcome drug resistance, we selectively knocked down BASP1 using shRNA targeting the 3′UTR of mRNA. Here, using a lentiviral approach, we generated stable clones expressing BASP1 shRNA in cisplatin-resistant OSCC lines (Fig. 2, *A* and *B*). We found no difference in BASP1 knockdown cells (KD) in terms of growth and cell death in comparison to parental control (NtshRNA) (Fig. S2, *A* and *B*). However, selective knockdown of BASP1 restores cisplatin-induced cell death in drug-resistant OSCC lines and patient-derived lines (PDC1), which was effectively rescued upon reconstitution of BASP1 expression (Fig. 2, *A*–*D*). PDC1 was isolated and characterized earlier from the tumor of a chemotherapy-non-responder patient, who was treated with neoadjuvant cisplatin and paclitaxel without any response (22). To validate our findings in a preclinical setup, we used Zebrafish (*Danio rerio*) (Tg(etsrp:GFP)) tumor xenograft model. We generated xenograft tumors using PDC1 NtshRNA, PDC1 BASP1 shRNA, as well as BASP1 (pCMV6 BASP1) overexpressing PDC1 cells in BASP1 KD condition, followed by cisplatin treatment. The tumor growth was found to be significantly reduced in cisplatin-treated BASP1 KD cells, which was rescued upon ectopic overexpression of BASP1 (Fig. 2, *E* and *F*).

### BASP1 is a major driver of the EMT phenotype in drug-resistant OSCC

**Figure 3:**
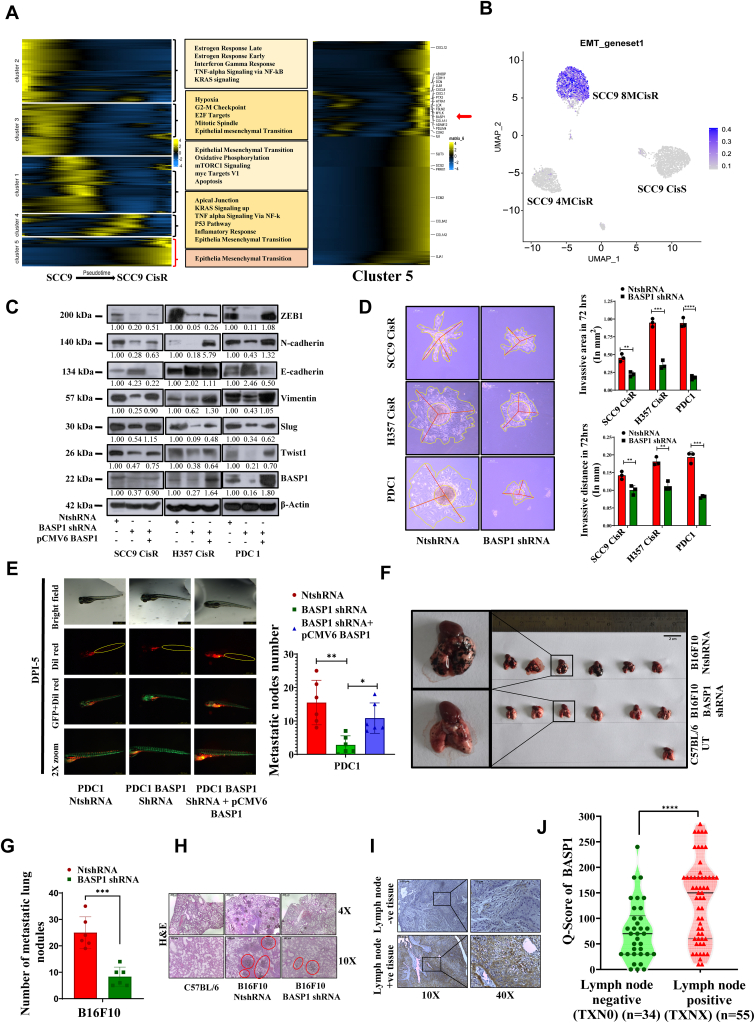
**BASP1-Mediated Regulation of EMT and Cell Migration.***A,* modelled gene expression values and their associated pathways across pseudotime from sensitive to resistance (*left to right*) for the cellular trajectory. A heatmap shows genes significantly associated with pseudotime (adjusted *p*-value ≤ 0.05, tradeSeq association test). Genes were clustered based on their expression patterns across pseudotime using Manhattan distance and hierarchical clustering with a 6-cluster cutoff. *B,* uniform manifold approximation and projection showing the expression of different EMT genes in three major clusters and transitional clusters. The intensity of *blue* colour represents the level of gene expression in individual cells, scaled separately for sensitive, early resistant and late resistant cell populations.*C,* BASP1 was transiently overexpressed in chemoresistant cells stably expressing BASP1 shRNA. After which lysates were isolated and immunoblotting (n = 3) was performed in cells expressing BASP1 NtshRNA, BASP1 shRNA and pCMV6 BASP1 using the indicated antibodies. *D, left panel*: Representative images of spheroids (in matrigel) derived from indicated cells at the indicated time points, demonstrating their invasive potential. Quantification of invasive area representing the outer rim of cells (in *yellow line*) and distance (in *red line*) from the center of spheroid. Images were captured using an inverted Leica DMIL microscope. Data represent the mean ± SEM of n = 3 independent experiments, ∗*p* ≤ 0.05. Statistical significance was determined by 2-way ANOVA. *Right panel*: Graph showing invasive area and invasive distance which was calculated using ImageJ (n = 3). *E, left panel*: Representative stereomicroscopic images of fluorescent transgenic Tg(etsrp:EGFP) zebrafish embryos xenografts at 5 days post-injection (dpi) with Dil-Red-stained PDC1 NtshRNA, BASP1 KD cells, and pCMV6 BASP1 over expression in BASP1 KD cells. Quantification of number of metastatic nodes observed in distal tail region was performed using ImageJ software (n = 6; mean ± SD). ∗*p* ≤ 0.05, ∗∗*p* ≤ 0.01 indicates statistically significant differences (2-way ANOVA). *Right panel*: Graph indicating number of metastatic nodes. *F,* representative lung images of mice injected (i.v.) with B16F10 cells stably expressing NtshRNA or BASP1 shRNA. *G,* graph shows the quantitative analysis of metastatic lung-nodules in mice from *panel F* (n = 6; mean ± SD). ∗∗∗*p* ≤ 0.0001. statistically significant differences (1-way ANOVA). *H,* hematoxylin and eosin (*H* and *E*) staining of different C57BL/6 UT, NtshRNA, and BASP1 shRNA groups at 4× and 10× magnification. *Red circles* indicate metastatic nodules in the lungs. *I,* representative images of immunohistochemistry staining for BASP1 in tumor tissue from OSCC (lymph node positive and negative) patient samples. Scale bar: 200 μm. *J,* immunohistochemistry scoring for BASP1 (Q score = staining intensity × percent of staining) was performed from *panel I* and plotted in a violin plot using graphpad prism for lymph node negative (n = 38) samples and lymph node positive (n = 55) tissue samples. ∗∗∗∗*p* ≤ 0.0001 by 2-tailed Student's *t* test. BASP1, Brain Abundant Membrane-Attached Signal Protein 1; OSCC, oral squamous cell carcinomas.

To know about the pathways that got deregulated in cisplatin-resistant OSCC lines, we performed Gene Ontology analysis from scRNA-seq data. Here, we found five different clusters based on the gene expression profile in the cell pattern trajectory from sensitive to late resistance, along with pseudotime (Figs. 3*A* and S3, *A*–*E*). Here, we found BASP1 to be in cluster 5, where EMT emerged as the only deregulated pathway (Fig. 3*A*). In this cluster, we found several genes related to EMT that were upregulated along with BASP1 (Fig. 3, *A* and *B*). Based on these observations, we assume that BASP1 might have a potential role in EMT transition during acquired drug resistance. To validate our hypothesis, we performed several assays to assess the role of BASP1 in EMT. Our immunoblotting data suggest that all major EMT transcription factors, like Slug, Twist1, and ZEB1, are significantly downregulated in BSAP1 KD cells, and their expression could be rescued upon reconstitution of BASP1. As a consequence of this, we found enhanced epithelial marker E-cadherin and reduced mesenchymal markers N-cadherin and vimentin in BASP1 KD, indicating mesenchymal-epithelial transition phenotype. However, when BASP1 was ectopically overexpressed in BASP1 KD cells EMT phenotype was found to be rescued (Fig. 3*C*). Next, we found that selectively knocking down BASP1 in cisplatin-resistant OSCC lines significantly reduced the migration, which was rescued after ectopic overexpression of BASP1 (Fig. S4, *A* and *B*). Further, we tested the BASP1 KD effect on invasion in a 3D model. Here, we first established the spheroids of NtshRNA and BASP1 shRNA cisplatin-resistant cells and then transferred them into matrigel for further growth. We found a significant reduction in invasive area and distance in BASP1 shRNA cells compared to NtshRNA stable clones (Fig. 3*D*), indicating mesenchymal-epithelial transition. Further to know in the *in vivo* context, we injected Dil-stained PDC1 NtshRNA and PDC1 BASP1 shRNA cells into the perivitelline space of 48-h post-fertilized zebrafish embryos. At post day 5 from injection, cancer cells showed reduced distal migration from the primary site in the case of the BASP1 KD group, which was rescued upon overexpression of BASP1 (Fig. 3*E*). Finally, we checked the metastatic activity of BASP1 in a mouse model. Mouse melanoma line B16F10 is known to form tumor nodules in the lungs of C57BL/6 mice when it is injected intravenously (i.v.). Here, our data suggests a significantly reduced number of metastatic lung nodules in the case of BASP1 shRNA cells as compared to NtshRNA stable clones of B16F10 (Fig. 3, *F* and *G*). The hematoxylin and eosin staining of paraffin-embedded tissue sections of lungs showed reduced metastatic nodules in BASP1 KD group (Fig. 3*H*). We also found that knocking down BASP1 in B16F10 results in the reduction of EMT markers, migration, and invasion property (Fig. S5, *A*–*E*). These data suggest that BASP1 is an important player for EMT in drug-resistant OSCC. To validate our findings in clinical samples, we scored the expression of BASP1 in OSCC patient tissues using immunohistochemistry. Our data showed significantly enhanced expression of BASP1 in lymph node-positive OSCC tumor tissues as compared to lymph node-negative tumor tissues (Fig. 3, *I* and *J* and Table S2). Overall, our data suggest that BASP1 is a key player of EMT in cisplatin-resistant cells.

### Transcriptome analysis revealed BASP1 regulates EMT through LIN7A-β-catenin axis:

**Figure 4:**
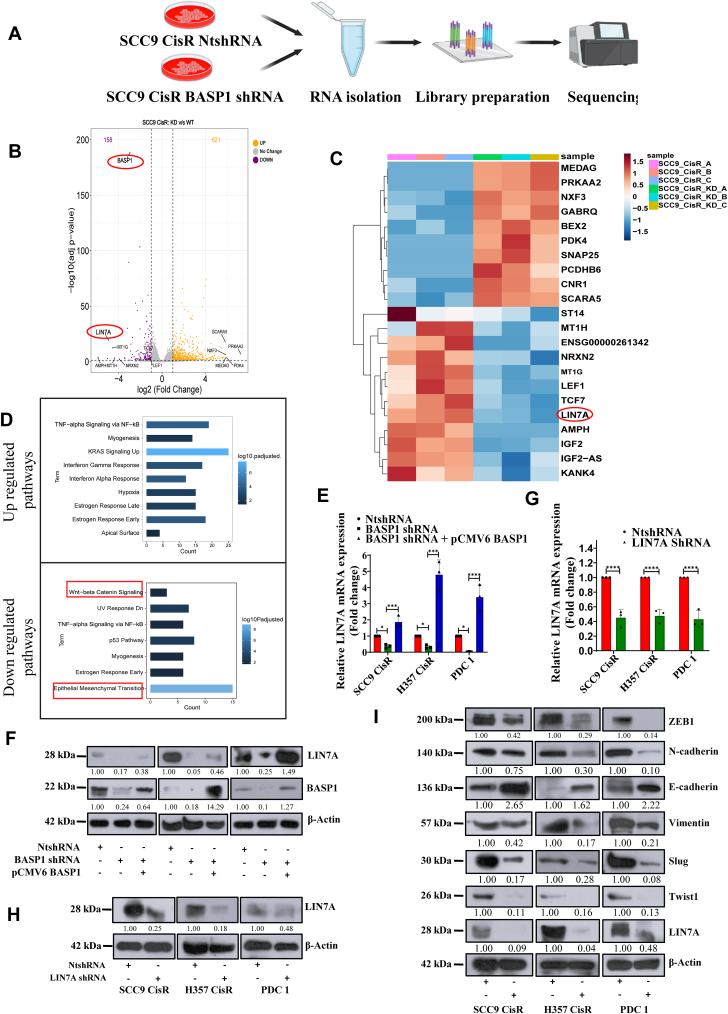
**Bulk RNA sequence reveals BASP1 regulates EMT *via* LIN7A.***A,* schematic representation of the bulk RNA-sequencing workflow for the indicated cell lines. *B,* bulk RNA sequencing was performed in SCC9CisR cells stably expressing NtshRNA and BASP1 shRNA cells (n = 3). Volcano plot showing differentially expressed genes between NtshRNA and BASP1 shRNA (BASP1 KD) cells. *C,* heat map of top differentially expressed genes between NtshRNA and BASP1 shRNA cells from bulk RNA sequencing data. *D,* pathway enrichment analysis was performed using the enrichGO function to identify significantly enriched pathways in differentially expressed genes between NtshRNA vs. BASP1 shRNA groups. *E* and *G,* relative mRNA expression of LIN7A, which was analyzed by quantitative real-time PCR (qRT-PCR) in the indicated cell lines. Data are presented as mean ± SEM (n = 3), ∗∗∗∗*p* ≤ 0.0001 by 1-way ANOVA. *F,* lysates were isolated from chemoresistant cells stably expressing NtshRNA, BASP1 shRNA, ectopic overexpression of (pCMV6 BASP1) and subjected to immunoblotting (n = 3) using indicated antibodies. *H* and *I,* lysates were isolated from chemoresistant cells stably expressing NtshRNA or LIN7A shRNA and subjected to immunoblotting (n = 3) using indicated antibodies.

**Figure 5:**
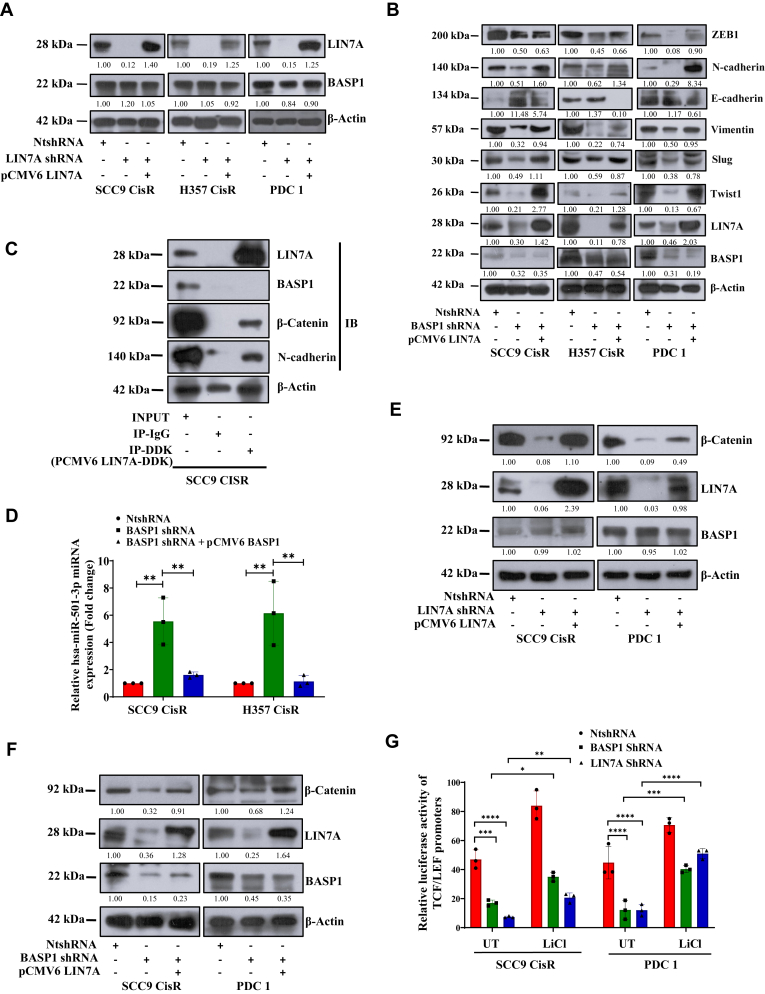
**BASP1-LIN7A axis regulates β-catenin signalling.***A,* lysates were isolated from chemoresistant cells stably expressing NtshRNA, LIN7A shRNA and ectopic over expression of LIN7A and subjected to immunoblotting (n = 3) using indicated antibodies. *B,* lysates were isolated from chemoresistant cells stably expressing NtshRNA, BASP1 shRNA and ectopic over expression of LIN7A (pCMV6 LIN7A) and subjected to immunoblotting (n = 3) using indicated antibodies. *C,* SCC9 CisR cells were transfected with pCMV6 LIN7A (DDK tagged). Lysates were then isolated, immunoprecipitated with anti-DDK antibody, and immunoblotted with indicated antibodies. *D,* relative miRNA hsa-miR-501-3P expression levels measured by quantitative PCR. Data are presented as mean ± SEM (n = 3). Statistical significance was determined by one-way ANOVA, ∗∗*p* ≤ 0.01. *E,* lysates were isolated from chemoresistant cells stably expressing NtshRNA, LIN7A shRNA and ectopic over expression LIN7A and subjected to immunoblotting (n = 3) using indicated antibodies. *F,* lysates were isolated from chemoresistant cells stably expressing NtshRNA, BASP1 shRNA and ectopic over expression LIN7A and subjected to immunoblotting (n = 3) using indicated antibodies. *G,* chemoresistant cells stably expressing NtshRNA, BASP1 shRNA or LIN7A shRNA were co-transfected with TOPflash and Renilla luciferase reporter vectors. Cells were treated with LiCl (20 mM) for 12 h, and luciferase activity was measured as described in the Methods section. The bar graph shows the relative luciferase activity in each group (n = 3, 2-way ANOVA), ∗*p* ≤ 0.05.

To understand the potential role of BASP1 in EMT, we compared the global gene expression profiles between BASP1 shRNA (KD) and non-targeting vector transduced (NtshRNA) chemoresistant cells (Fig. 4*A*). In our transcriptomics data, we found 779 deregulated genes (FDR < 0.05), out of which 621 genes found to be upregulated (>2 fold) and 158 to be downregulated (<2fold) (Fig. 4*B*). The heatmap of top 10 deregulated genes showed Lin-7 Homolog A, Crumbs Cell Polarity Complex Component (LIN7A) as one of the most downregulated gene in BASP1 KD cells (Fig. 4*C*). Similarly, we performed Gene Ontology analysis to explore deregulated pathways. As expected, we found EMT pathways to be downregulated in BASP1 KD cells. In addition to this, we also found Wnt-β-catenin signalling to be downregulated in BASP1 KD cells (Fig. 4*D*). To validate the finding from RNA-Seq, we performed immunoblotting and qRT-PCR. Our data suggest that LIN7A expression is significantly downregulated in BASP1 KD cells, and it was efficiently rescued by ectopic overexpression of BASP1 (Fig. 4, *E* and *F*). To explore if LIN7A regulates EMT, we generated clones of chemoresistant OSCC cells stably expressing LIN7A shRNA and NtshRNA (Fig. 4, *G* and *H*). Our immunoblotting data suggests that selectively knocking down LIN7A in drug resistant OSCC results in suppression of EMT phenotype (Fig. 4*I*). We found no change in BASP1 expression in LIN7A KD clones indicating LIN7A is a downstream target of BASP1 (Fig. 5*A*). Further to know if LIN7A is downstream mediator of BASP1 induced EMT, we ectopically overexpressed LIN7A in BASP1 KD cells and performed immunoblotting to score EMT. The data clearly demonstrate that overexpression of LIN7A efficiently rescued the BASP1 KD-mediated reduction of EMT phenotype in cisplatin-resistant OSCC (Fig. 5*B*). We performed an immunoprecipitation assay and found that LIN7A interacts with β-catenin and N-cadherin but not with BASP1 (Figs. 5*C* and S6, *A* and *B*). Our analysis did not reveal any direct interaction between BASP1 and LIN7A. Hence, we performed a literature survey to find the missing link between BASP1 and LIN7A. Chubin Luo *et al.*, in their investigation, showed a strong negative correlation between miR-501-3p and LIN7A expression. They further went on to confirm that LIN7A is a functional downstream effector of microRNA miR-501-3p in promoting hepatocellular carcinoma (HCC) progression (23). We hypothesized that BASP1 regulation of LIN7A might occur through miR-501-3p. Our qRT-PCR observation revealed that BASP1 inversely regulates the microRNA hsa-mir-501-3p which in turn augments the expression of LIN7A mRNA (Fig. 5*D*). Overall, these data prompted us to assume that LIN7A might regulate Wnt-β-catenin signalling and hence EMT in BASP1 expressing resistant cancer cells. The immunoblotting data showed a substantial downregulation of β-catenin in LIN7A KD cells as compared with control cells (Fig. 5*E*). Similarly, we found downregulation of β-catenin in BASP1 KD cells which can be rescued by overexpression of LIN7A (Fig. 5*F*). In addition to this, stable BASP1 KD or LIN7A KD chemoresistant cells depicted significantly reduced TOPflash luciferase activity, indicating diminished β-catenin/TCF-LEF–mediated transcriptional activity (Fig. 5*G*). These data indicates that BASP1 regulates LIN7A-mediated Wnt-β-catenin signalling. It is known from the literature that Wnt-β-catenin signalling augments EMT and metastasis (24).

### BASP1 regulates LIN7A expression through AKT/glycogen synthase kinase

**Figure 6:**
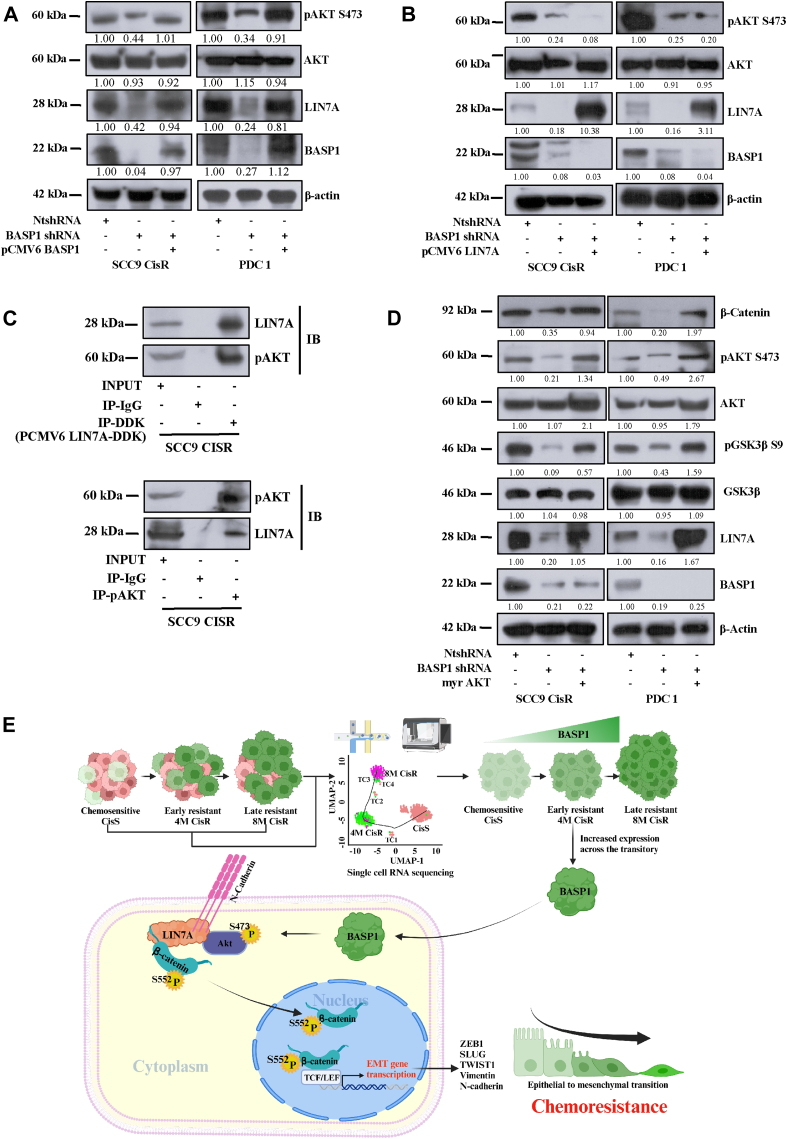
**BASP1 regulates EMT through RAC-alpha serine/threonine-protein kinase (AKT)/β-catenin axis.***A,* lysates were isolated from chemoresistant cells stably expressing NtshRNA, BASP1 shRNA or ectopic overexpressing BASP1 (pCMV6 BASP1). Immunoblotting (n = 3) was performed using indicated antibodies. *B,* lysates were isolated from chemoresistant cells stably expressing NtshRNA, BASP1 shRNA, or overexpressing LIN7A (pCMV6 LIN7A) in BASP1 shRNA cells. Immunoblotting (n = 3) was performed using indicated antibodies. *C,* SCC9 CisR cells were transfected with pCMV6 LIN7A (DDK tagged) or Myr-AKT (constitutively active AKT). Lysates were then isolated, immunoprecipitated with anti-DDK antibody for LIN7A and p-AKT and immunoblotted with indicated antibodies. *D,* Myr-AKT was overexpressed in cells stably expressing BASP1 shRNA and lysates were isolated from chemoresistant cells and subjected to immunoblotting (n = 3) using indicated antibodies. *E,* schematic representation of the mechanism by which BASP1 regulates EMT *via* LIN7A in cisplatin resistant OSCC. BASP1, Brain Abundant Membrane-Attached Signal Protein 1; EMT, epithelial to mesenchymal transition; OSCC, oral squamous cell carcinomas.

Since we did not find any interaction between BASP1 and LIN7A, we explored how BASP1 expression correlates with the regulation of LIN7A. In the literature, we found that in lung cancer cells, selective knockdown of BASP1 results in decreased phosphorylation of RAC-alpha serine/threonine-protein kinase (AKT) (25). Hence, we performed immunoblotting and found that pAKT S473 expression was significantly reduced in BASP1 KD cells which was efficiently rescued with ectopic expression of BASP1 (Fig. 6*A*). However, when LIN7A was overexpressed in BASP1 KD cells, we did not find any increase in pAKT S473 expression indicating LIN7A is downstream to AKT (Fig. 6*B*). Further our immunoprecipitation data suggests that LIN7A interacts with p-AKT (Fig. 6*C*). Moreover, the myr-AKT overexpression (constitutively active AKT) in BASP1 KD cells efficiently rescued p-AKT S473, p-GSK3β S9 and β-catenin expression (Fig. 6*D*). Interestingly, we also found that myr-AKT overexpression in BASP1 KD cells, rescued LIN7A expression indicating AKT might stabilize LIN7A expression (Fig. 6*D*).

## Discussion

A few cells within the isogenic tumor mass may show differences in gene expression profiles in response to intrinsic or extrinsic factors (26). This phenomenon is termed gene expression noise, which results in phenotypic heterogeneity in a small population of cells in the tumor mass. This phenotypic heterogeneity is believed to contribute to intratumor heterogeneity. Driven by expression noise in the tumor mass, a rare population of cells can escape the cytotoxic activity of chemotherapy drugs, which contributes to drug resistance, continued tumor growth, and relapse (27). EMT is a phenomenon that is believed to be initiated by expression noise. The EMT is primarily driven by the EMT transcription factors that include SNAIL, SLUG, TWIST1, ZEB1, and ZEB2. These transcription factors reduce the hallmark epithelial E-cadherin and activate the mesenchymal phenotype like N-cadherin, vimentin, and fibronectin. The classical role of EMT in tumor tissues is to augment invasion by reducing the epithelial integrity that results in the dissemination of cancer cells from tissues, which is the first step of the metastasis process. However, the non-classical roles of EMT in cancer cells involve acquiring drug resistance, enhancing stemness, and metabolic reprogramming (28). In breast cancer cells, docetaxel induced Twist binds to the promoter of ABC transporters. This led to increased expression of ABC expression resulting EMT and EMT-induced multidrug resistance (15). In lung cancer cells, AKT/NF-κB induced increased slug expression blocks the proapoptotic BH3-like proteins p53 upregulated modulator of apoptosis, resulting in cisplatin resistance (29). In addition to this, overexpression of slug and SNAIL led to inactivation of tumor suppressor genes p53 and PTEN, antagonizing their pro-apoptotic activity, resulting in drug resistance (30). Similarly, EMT plays an important role in enhancing stemness in cancer cells, and that is how it contributes to drug resistance. The EMT transcription factor Twist1 directly regulates stemness factor Bmi1 which represses expression of both E-cadherin and p16INK4a in head and neck squamous cell carcinomas that led to EMT-induced self-renewal and tumor-initiating activity (31). Similarly, ZEB1 plays an important role in enhancing Notch signalling-mediated stemness and hence drug resistance in pancreatic and breast carcinomas (32). Overall, these studies suggest that EMT is a major contributor to cancer stemness and acquired drug resistance in various neoplasms. In this study, we performed single-cell RNA sequencing of human OSCC lines presenting with sensitive, early, and late cisplatin-resistance patterns to identify the potential role of rare population of cells in chemoresistance. Our data suggests the existence of two different rare molecular subtypes of cells, which are termed as transitional clusters (TC1-TC2). From differential gene expression data, we found BASP1 to be upregulated not only in two major drug-resistant (early and late) but also in two major transitional clusters. Pathway analysis showed that BASP1 might play an important role in EMT. Here, we found that BASP1 is a major player in EMT-induced cisplatin-resistant OSCC. Here, we anticipate that BASP1-induced EMT augments the acquired cisplatin resistance in OSCC lines. Selective knockdown of BASP1 reverses EMT phenotype in cisplatin-resistant cells and restores cisplatin-induced cell death.

To explore the mechanism by which BASP1 regulates EMT, we performed bulk RNA sequencing between BASP1 shRNA and control transduced (NtshRNA) chemoresistant cells. Here, we found LIN7A to be significantly downregulated in BASP1 KD cells. In hepatocellular carcinomas (HCC), LIN7A augments EMT and metastasis. In HCC lines, microRNA miR-501-3p targets and degrades LIN7A to inhibit N-cadherin, vimentin, and SNAIL (23). Ectopic overexpression of LIN7A enhanced the invasion phenotype of breast carcinomas (33). However, the role of LIN7A in the EMT of OSCC is not yet known. Our data suggest that BASP1 positively regulates LIN7A to augment EMT in cisplatin-resistant cells. Our immunoprecipitation data suggest that BASP1 does not interact with LIN7A. In the literature, we found that in lung cancer cells, selective knockdown of BASP1 results in decreased phosphorylation of AKT (25). Our immunoprecipitation data suggest that AKT interacts with LIN7A and hence stabilizes its expression in drug-resistant cells. Further, LIN7A interacts with β-catenin to augment Wnt-signalling-driven EMT.

Using scRNA-seq, we found two different rare molecular subtypes of cells (TC1-TC2) that contribute to the phenotypic heterogeneity among isogenic OSCC lines. Analysing the gene expression profiles and deregulated pathways of scRNA-seq data, we found that BASP1 is a key regulator of EMT in acquired cisplatin-resistant cells. Here, the EMT phenotype is primarily responsible for acquiring drug resistance in OSCC. A complete representation of the study is outlined in Figure 6*E*.

## Experimental procedures

### Cell culture:

OSCC lines H357 and SCC9 (obtained from ECACC) and the chemotherapy-non-responder patient-derived PDC1 cells, were cultured in DMEM/F-12 (Thermo Fisher Scientific, Cat No# 12500062) media and with 0.6 μg/ml sodium hydrocortisone (Sigma, Cat No# H0135). PDC1 was isolated and characterized earlier from the tumor of a chemotherapy-non-responder patient, who was treated with neoadjuvant cisplatin and paclitaxel without any response (21). HEK 293 T and B16F10 (Obtained from Cell repository, NCCS), were routinely maintained in DMEM media (Thermo Fisher Scientific, Cat No# 12800017). All cells were cultured and maintained supplemented with 10% FBS (Gibco, Cat No# A5256701), 1% penicillin-streptomycin (Himedia, Cat No# A001) in a humidified atmosphere of 5% CO_2_.

### Generation of cisplatin-resistant cell line

Generation of acquired resistant cell lines were carried out from the original parental cell lines through continuous exposure to chemotherapeutic drugs. MTT (3-(4,5-dimethylthiazol-2-yl)-2,5-diphenyltetrazolium bromide) assay was used to determine the drug sensitivity by measuring the IC50 doses of cisplatin in SCC9 and H357 cell lines. Initially, cells were exposed to gradually increasing drug concentrations up to their respective IC50 values as described by Maji S *et al.*, 2019, (22). Following this, cells were continually cultured for 8 months in the presence of these IC50 concentrations until they regained a normal growth pattern. During this resistant cell lines generation by around the fourth month, those cell lines start showing the resistant phenotypes, which we have termed as early resistant cells (H357 4MCisR and SCC9 4MCisR). Similarly, after 8 months, we termed them as late resistant cell lines (H357 8MCisR and SCC9 8MCisR).

### Single-cell RNA sequencing

Single-cell RNA sequencing was performed with SCC9 CisS, SCC9 4MCisR and SCC9 8MCisR lines using the 10× Genomics’ single cell RNA-seq (scRNA-seq) technology according to the manufacturer’s protocol. Briefly, cells were harvested, counted by trypan blue exclusion, hash-tagged using 1 μg of TotalSeq C hashtag antibody from Biolegend as per recommendation and loaded along with reverse transcription (RT) reagents, Gel Beads containing barcoded oligonucleotides, and oil on a microfluidic chip to form reaction vesicles called Gel Beads in Emulsion (GEMs) using the Chromium Controller instrument (10× Genomics). Each functional GEM contained a single cell, a single gel bead, and RT reagents. Further, each cell got lysed, the gel bead dissolved to free the identically barcoded RT oligonucleotides into solution, and reverse transcription of polyadenylated mRNA occurred within each GEM. Thus, all cDNAs from a single cell had the same barcode, allowing the sequencing reads to be mapped back to their original single cell of origin. Next, 5ʹ Gene expression library and cell surface protein library construction was performed from these barcoded cDNAs using Chromium Next GEM Single Cell 5′ Reagent Kits v2 according to the manufacturer’s protocol, followed by library concentration estimation using Qubit 4.0 (Invitrogen) and the recommended fragmentation size confirmation by Bio-analyzer (Agilent). Finally, prepared libraries were sequenced using Illumina NextSeq 550 platform.

### Single-cell RNA data preprocessing

The Cell Ranger mkfastq (v5) from 10× Genomics was used to demultiplex raw binary base call (BCL) files to fastq files. The generated fastq files were then aligned to the human reference genome (refdata-cellranger-GRCh38–3.0.0) using cellranger multi. 10× feature barcode information was included in the feature reference. The resulting output generated was a gene expression matrix and feature barcode counts for each cell barcode. The matrix was then processed in R (v4.1) using the Seurat package (v4.3) (34). As a primary quality control step, we first filtered out cells with < 300 expressed genes. To filter out potential doublets, we have discarded cells with a total number of detected genes > 4000. Furthermore, we have calculated the percentage of mitochondrial genes expressed and discarded cells with more than 5% mitochondrial genes of all the detected cells. Finally, the hashtag samples were demultiplexed using the HTO demux pipeline, which resulted in a total of 4356 cells. The three samples were normalized for cell counts using Sctransform (35) method.

### Dimension reduction and clustering

We used principal component analysis (PCA) to reduce the dimensionality of the dataset. Next, we performed unsupervised clustering as implemented in Seurat (34). We first constructed a shared k-nearest neighbor graph based on the euclidean distance in PCA space, using 10 PCA components and clustered the cells using the default algorithm. We visualized the clusters by uniform manifold approximation and projection. Using the default non-parametric Wilcoxon rank-sum test, we determined the marker for each of the clusters.

### Trajectory analysis

To understand the dynamic gene expression pattern from sensitive to resistant cells, we used slingshot (36) to infer cell trajectory and pseudotime. The algorithm first constructs trajectories by building a minimum spanning tree and then fits smooth curves through the data for each of the identified trajectories and assigns each cell a pseudotime value along these trajectories. First, we converted the Seurat object to Single Cell Expriment (37) object and used the slingshot function to perform trajectory analysis. Next, to identify the genes whose expression levels vary with pseudotime, we have used the tradeSeq (38) algorithm. For each gene, we fitted a general additive model to model the relationships between gene expression and pseudotime. Finally, to test for significant associations between expression and pseudotime, we used the associationTest function with default parameters.

### Library preparation for bulk RNA-Sequencing

RNA-Seq library preparation was performed for SCC9 CisR NtshRNA and SCC9 CisR BASP1 shRNA stable lines. Three independent biological replicates were included to identify the BASP1 depletion-mediated global transcriptome changes. First, 500 ng of total RNA was used to isolate mRNA through magnetic beads using mRNA isolation kit (PolyA mRNA Isolation Module, NEB #E7490) followed by RNA-Seq library preparation using mRNA library preparation kit (NEB #E7770) according to the vendor’s recommended protocol. After library preparation, library concentration was estimated using Qubit 4.0 (Invitrogen), the recommended fragmentation sizes were confirmed by Bio-analyzer (Agilent) and libraries were sequenced using Illumina NextSeq 550 platform.

### RNA-seq data processing and analysis

Raw reads of BASP1 KD RNA-seq samples and their matched control were checked for quality using FASTQC and then aligned to the human genome (UCSC hg38) using hisat2 (version 2.2.1) (39) (with default parameters). Raw counts were extracted from the respective samples using the featureCounts tool (version 2.0.1) (40). These counts were further analyze for differential gene expression analysis using DESeq2 (version 1.30.1) (41). Differentially expressed genes were filtered based on fold change (upregulated ≥ 2 and downregulated ≤ −2) and adjusted *p*-value < 0.05. Principal component analysis was performed on variance stabilized transformed values using the plotPCA function and plotted using ggplot2.

### Functional enrichment analysis

Gene ontology over-representation analyses were performed in R (version 4.1) using the enrichGO function in the clusterProfiler (version 3.18.1) (42) package. Adjusted *p*-value < 0.05 was used to filter out significantly enriched biological processes. All the figures are plotted in R using the ggplot2 (version 3.5.1) package, and for the heatmap, the ComplexHeatmap (version 2.8.0) (43) package is used.

### Lentivirus production and generation of stable BASP1-KD and LIN7A-KD cell lines

Following the Addgene protocol, shRNAs that target 3′UTR region of BASP1 and LIN7A were cloned into the pLKO.1 vector individually. Sanger sequencing confirmed the successful shRNA cloning. pLKO.1 shRNA plasmid was transfected along with plasmid pCMV delta R8.2 packaging and plasmid pMD2G envelope into HEK293 T cells seeded at approximately 40 to 60% confluency. Following transfection, the cells were maintained for 48 h to facilitate lentivirus production. Using the procedure outlined in Shriwas *et al.* (44)**,** lentivirus particles were isolated. Lentivirus-infected cells were treated with puromycin (Gibco, Cat No# A11138–03) at concentrations up to 5 μg/ml for 2 weeks to select stable clones, and knockdown was confirmed by immunoblotting. ShRNA sequences used in this investigation are listed in Table S01.

### Transient transfection and overexpression of BASP1, LIN7A, as well as myr-AKT

Stable cell lines SCC9 CisR, H357 CisR and PDC1 expressing shRNA targeting the 3′UTR region of BASP1 mRNA and LIN7A mRNA, respectively, were generated. These cell lines were then transiently transfected with pCMV6-BASP1 (Myc-(DYKDDDDK) DDK-tagged) (Origene, Cat No# RC201815) and pCMV6-LIN7A (Myc-DDK-tagged) (Origene, Cat No# RC221902) using ViaFect transfection reagent (Promega, Cat No# E4982) separately at approximately 40 to 60% confluency. Successful transfection was confirmed by immunoblotting with anti-BASP1 and anti-LIN7A antibodies, respectively. To overexpress AKT, 901 pLNCX-myr-HA-Akt1 (Addgene plasmid # 9005) was transiently transfected into target cells. 901 pLNCX myr HA Akt1 was a kind gift from William Sellers (45).

### OSCC patient sample

OSCC patient tumor tissues were collected from All India Institute of Medical Sciences, and staged according to the American Joint Committee on Cancer (AJCC) tumor, node, metastasis staging system. Patients were classified as lymph node-negative (N0) if no lymph node metastasis was detected, and lymph node-positive (Nx) if nodal metastasis was present. All patient-related studies were approved by the Human Ethics Committee (HEC) of the Institution of Life Sciences, and informed consent was obtained from all participants. Detailed information on study subjects, including lymph node status and tumor, node, metastasis stage, is provided in Table S02, *A* and *B*.

### Immunoblotting

Cell lysates were prepared as described previously (21) and subjected to immunoblotting. For transient overexpression experiments, cells were seeded at 40% confluency and transfected with the respective expression plasmid vectors using ViaFect transfection reagent (Promega, Cat No# E4982). The transfected cells were then cultured for 48 h, followed by lysate isolation as per the user manual. For this study, we employed antibodies against β-actin (MilliporeSigma, Cat No# A2066), BASP1 (Sigma, Cat No# HPA045218), BASP1 Polyclonal (Mybiosources Cat No# MBS9612337), LIN7A-(Abcam, Cat No# ab174297), TWIST (CST, Cat No# 69366S), Slug (CST, Cat No# 9858S), Vimentin (CST, Cat No# 5741S), E-cadherin (CST, Cat No# 14472S), N-cadherin (CST, Cat No# 13116S), ZEB1 (CST, Cat No# 70512S) AKT (CST, Cat No# 9272S), p-AKT (S473) (CST, Cat No# 4058S), GSK-3β (CST, Cat No# 9315s), p-GSK-3β (S9) (CST, Cat No# 9323S), β-catenin (CST, Cat No# 9562).

### Co-immunoprecipitation

SCC9 CisR cell line was lysed in 1% RIPA (CST, Cat No# 9806S) for 30 min on ice to solubilize proteins while maintaining protein-protein interactions. Then, 1 μg of total protein lysate was incubated with specific immunoprecipitation (IP) grade primary antibody for the protein of interest overnight with gentle rotation at 4 °C to allow antibody-antigen complex formation. Subsequently, Dyna beads, Protein-G (Invitrogen Thermo Fisher Scientific, Cat No# 10004D), were added to the lysate and incubated for 1 to 4 h at 4 °C. The samples were washed four times with 1% RIPA to remove unbound proteins. Bound proteins were then eluted from the beads using SDS sample buffer and heated at 95 °C for 5 min. The eluted proteins were separated by SDS-PAGE and analyzed by immunoblotting. For this study, we used IP grade antibodies DYKDDDDK Tag (D6W5B) (CST, Cat No# 14793S) and for p-AKT (S473) (CST, Cat No# 4058S). The proteins of interest and their interacting partners were detected using VeriBlot for IP Detection Reagent (HRP) (Abcam, Cat No# ab131366).

### RT-PCR and qRT-PCR

Total RNA was isolated from different cell lines as mentioned in figure using an RNA mini kit (Himedia, Cat No# MB602) and quantified using a NanoDrop spectrophotometer (Thermo Fisher Scientific). cDNA was synthesized from 500 ng of total RNA using the Verso cDNA synthesis kit (Thermo Fisher Scientific, Cat No# AB1453 A). Quantitative real-time PCR (qRT-PCR) was performed using SYBR Green master mix (Thermo Fisher Scientific, Cat No# 4367659) with 18S rRNA as a reference gene. The primer sequences (oligonucleotides) used for qRT-PCR are listed in Table S01.

### Immunohistochemistry:

Formalin-fixed, paraffin-embedded tissue sections from OSCC patient tumors were subjected to immunohistochemistry staining as previously described (21) using antibodies against BASP1 (Sigma, Cat No# HPA045218). Stained tissue sections were imaged using a Leica DM500 microscope. To quantify protein expression, a Q-score was calculated for each protein, which is a product of the percentage of positively stained cells (P) and the intensity of staining (I). The intensity of staining was assessed on a scale of 0 to 3, where 0 represents no staining, one represents weak staining, two represents moderate staining, and three represents strong staining.

### Annexin V PE/7AAD assay

Apoptosis and cell death were assessed using the Annexin V Apoptosis Detection Kit PE (eBioscience, Cat No# 88–8102–74) and flow cytometry (BD FACS Fortessa), as described in the manufacturer’s protocol.

### Assessment of cell viability

OSCC cells were seeded at approximately 2000 cells per well in a 96-well plate and incubated for 48 h at 37 °C in a CO_2_ incubator following drug treatment. Cell viability was assessed using the MTT (3-(4,5-dimethylthiazol-2-yl)-2,5-diphenyltetrazolium bromide) assay (Sigma-Aldrich). For MTT assay, 0.5 mg/ml of MTT reagent was added to each well and incubated for 3 to 4 h 37 °C. The formed formazan crystals were dissolved in DMSO, and absorbance was measured at 490 nm by following the manufacturer's protocol.

### Zebrafish xenograft

All animal experiments were approved by the Institutional Animal Ethics Committee. The experiment was performed in accordance with the protocol as described by Ansari *et al.* (46). Briefly, PDC1/B16F10 control and BASP1-knockdown cells were DiI-labeled (Vybrant DiI, Cat# V22885), and ∼400 cells were microinjected into the perivitelline space of 48 hpf Tg(etsrp:GFP) zebrafish embryos. Embryos were imaged on Day 0, treated with cisplatin (100 ng/ml) on Day 3 where applicable, and imaged again on Day 5. Tumor burden was quantified as the change in fluorescence intensity (Day 5 vs Day 0) using ImageJ. For the metastasis assay, the same injection procedure was followed, and metastatic foci were counted on Day 5 (n = 6).

### *In vivo* mice metastasis assay

C57BL/6 mice (6–8 weeks old, male) were housed under specific pathogen-free conditions. All experimental procedures were approved by the Animal Ethics Committee of the Institute of Life Sciences and were conducted in accordance with approved guidelines and regulations. To study lung metastasis, control and BASP1 shRNA stably transfected B16F10 cells were resuspended in PBS at a concentration of 5 × 10^5^ cells/100 μl and intravenously injected into the lateral tail vein of C57BL/6 mice (n = 6 per group). After 3 weeks, mice were euthanized, and their lungs were harvested. The B16F10 tumor specimens were imaged for surface nodules count and later fixed in 10% formalin, embedded in paraffin, sectioned at 4 μm, and subjected to H and E staining.

### Trans-well invasion assay

B16F10 control and BASP1 stable knockdown cells were seeded in the upper chamber of a Cell Culture Inserts (Falcon, Cat No# 353097) plate containing 200 μl of serum-free medium, with $5\times 10^{4}$ cells for the invasion assay. The lower chamber was filled with 0.6 ml of DMEM media supplemented with 10% FBS. For invasion assay, the upper chamber of the transwell plate was coated with 4 mg/ml Matrigel Matrix (Corning, Cat No# 356234) only. After a 24-h incubation period, cells that had migrated through the membrane to the lower surface were fixed, stained with 0.6% crystal violet (SRL, Cat No# 28376), and visualized and were imaged using a Leica DM500 microscope, with each experiment performed in triplicate.

### *In vitro* scratch assay

Cells were allowed to reach approximately 80 to 90% confluency, after which an *in vitro* scratch assay was performed by creating a linear wound across the cell monolayer using a pipette tip. Detached cells were removed by gentle washing with PBS, and fresh culture medium was added to the wells to support continuing cell migration and wound closure. Cell migration into the scratch area was then quantified by imaging at different time points using ImageJ (https://imagej.net/ij/).

### MicroRNA isolation, cDNA synthesis, and qRT-PCR

MicroRNA was isolated using the magsure miRNA isolation kit (RNA Bio, Cat No# MAR50) according to the manufacturer’s user manual. RNA purity and concentration were assessed using a NanoDrop spectrophotometer (Thermo Fisher Scientific). Complementary DNA (cDNA) first strand was synthesized from the isolated miRNA using the mir-X-miRNA first strand synthesis kit (Takara, Cat No# 638313) following the manufacturer’s instructions followed by qRT-PCR run using primer U6 as a control and primer against microRNA hsa-501-3p as our target.

### Dual luciferase reporter assay

OSCC cell lines were co-transfected with the M50 Super 8× TOPFlash reporter plasmid (M50 Super 8× TOPFlash was a gift from Randall Moon (Addgene, plasmid # 12456) (47) and the pRL-TK Renilla (Promega, Cat No# E2241) luciferase control plasmid at a 100:1 ratio using ViaFect transfection reagent. The transfection was performed in white 96-well plates having 2000 cells in each well, and the cells were incubated for 48 h post-transfection with the indicated plasmid constructs. The TOPFlash reporter, containing seven TCF/LEF binding sites upstream of a firefly luciferase gene, enables measurement of Wnt-induced transcriptional activity. After 48 h of post-transfection, cells were treated with either vehicle control or LiCl (Wnt signalling activator) (Sigma, Cat No# 62476), in wild-type NtshRNA and BASP1 or LIN7A shRNA cell lines. Luciferase activity was subsequently measured using the Dual-Glo Luciferase Assay Kit (Promega, Cat No# 1910), with Renilla luciferase activity serving as an internal control to normalize for transfection efficiency.

### Tumor spheroid invasion assay

A total of 1000 OSCC cells were seeded on 6-well ultralow attachment plates (Corning, Cat No# 3471) and were grown with 1× B27 (Invitrogen, Cat No# 17502048), 1× N2 supplement (Invitrogen, Cat No# 17502048), 20 ng/ml human recombinant epidermal growth factor (Invitrogen, Cat No# PHG0313) and 10 ng/ml basic fibroblast growth factor (Invitrogen, Cat No# PHG0263) in serum-free DMEM-F12 medium (Pan biotech, Cat No# P04–41500). Spheroid formation was monitored with inverted microscope (LEICA DMIL). For invasion assay, Spheroids of wild-type NtshRNA and BASP1 shRNA cells were cultured in Matrigel under serum-free conditions. Representative images of invasive cells upon treatment (n = 3 independent experiments with multiple spheroids) are shown. Invasive area representing the outer rim of cells (see yellow lines), and invasive distance representing the mean distance covered by the 10 to 15 most invasive single cells, were quantified using ImageJ. Mean and SD are shown in scatter dot plots of n = 3 independent experiments, where each dot represents one spheroid.

### Statistics

All data are presented as mean ± SD and were analyzed using GraphPad Prism 8.4 (https://www.graphpad.com/). Statistical significance was determined by one-way and two-way ANOVA, with *p* ≤ 0.05 considered significant.

## Data and materials availability

scRNA-seq of OSCC cell line SCC9 (Cisplatin sensitive CisS, early resistant CisR4M, and late resistant CisR8M) and bulk RNA seq of BASP1 wildtype and knocked down Cisplatin resistant OSCC cell line (SCC9CisR) has been updated in Array Express, and the assigned ArrayExpress accession is “E-MTAB-15148” and “E-MTAB-15141” respectively. All data are available in the main text or the supplementary materials.

## Ethical approval

This study was approved by the Institutional Human Ethics Committee (146/HEC/2025) of ILS and the All India Institute of Medical Sciences (AIIMS) (T/EMF/Surg. Onco/19/03). Animal experiments adhered to protocols approved by the Institutional Animal Ethics Committee (IAEC) (ILS/IAEC-290-AH/NOV-22) of ILS. Patient consent was obtained, and tissue samples were collected as per approved procedures. All human studies reported in this manuscript were conducted in accordance with the principles embodied in the Declaration of Helsinki.

## Supporting information

This article contains supporting information.

## Conflict of interest

The authors declare that they have no conflicts of interest with the contents of this article.
