# Supplementary material for: Single-cell analysis identifies BASP1 as a driver of drug resistance and cell plasticity in oral squamous cell carcinoma
Source: J Biol Chem. 2026 Jan 6;302(2):111126. doi: 10.1016/j.jbc.2025.111126 (PMC12860937; doi:10.1016/j.jbc.2025.111126)
Supplement: Supporting information [file mmc1.pdf]

Supplementary figure 1

A

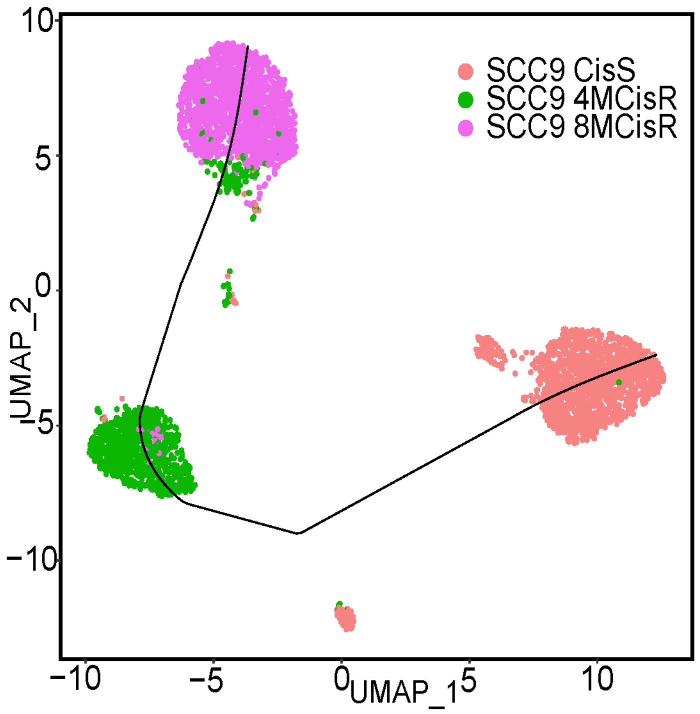

B

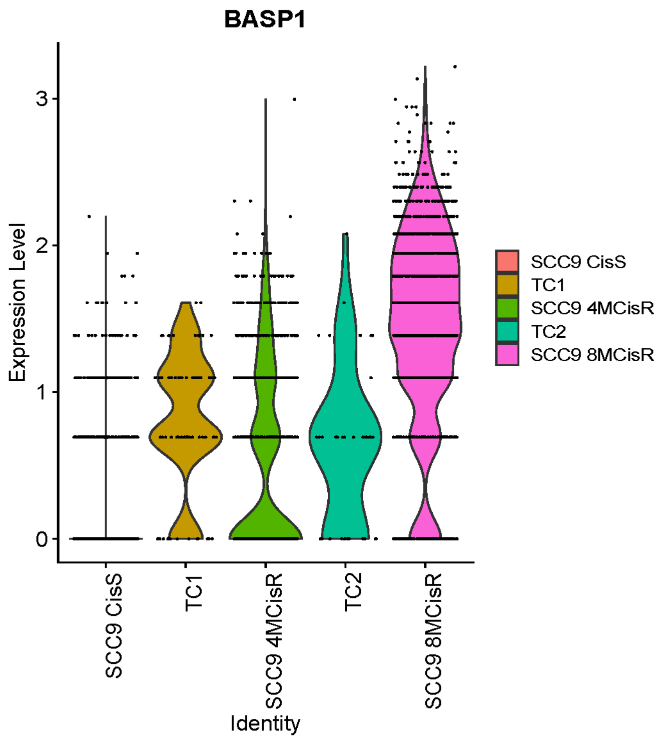

C

CELLS	COUNT
ESTIMATED NUMBER OF CELLS	4356
MEAN READ PER CELL	60596
MEDIAN GENES PER CELLS	2709
TOTAL GENE DETECTED	25151
MEDIAN UMI COUNTS PER CELL	9283

D

Clusters

Cell types

No. of cells	SCC9-CisS	TC1	SCC9-4MCisR	TC2	SCC9-8MCisR	Total CELLS
SCC9-CisS	1541	75	2	13	0	1631
SCC9-4MCisR	0	5	1014	28	0	1047
SCC9-8MCisR	0	0	5	0	1673	1678
TOTAL CELLS	1541	80	1021	41	1673	

Supplementary figure 2

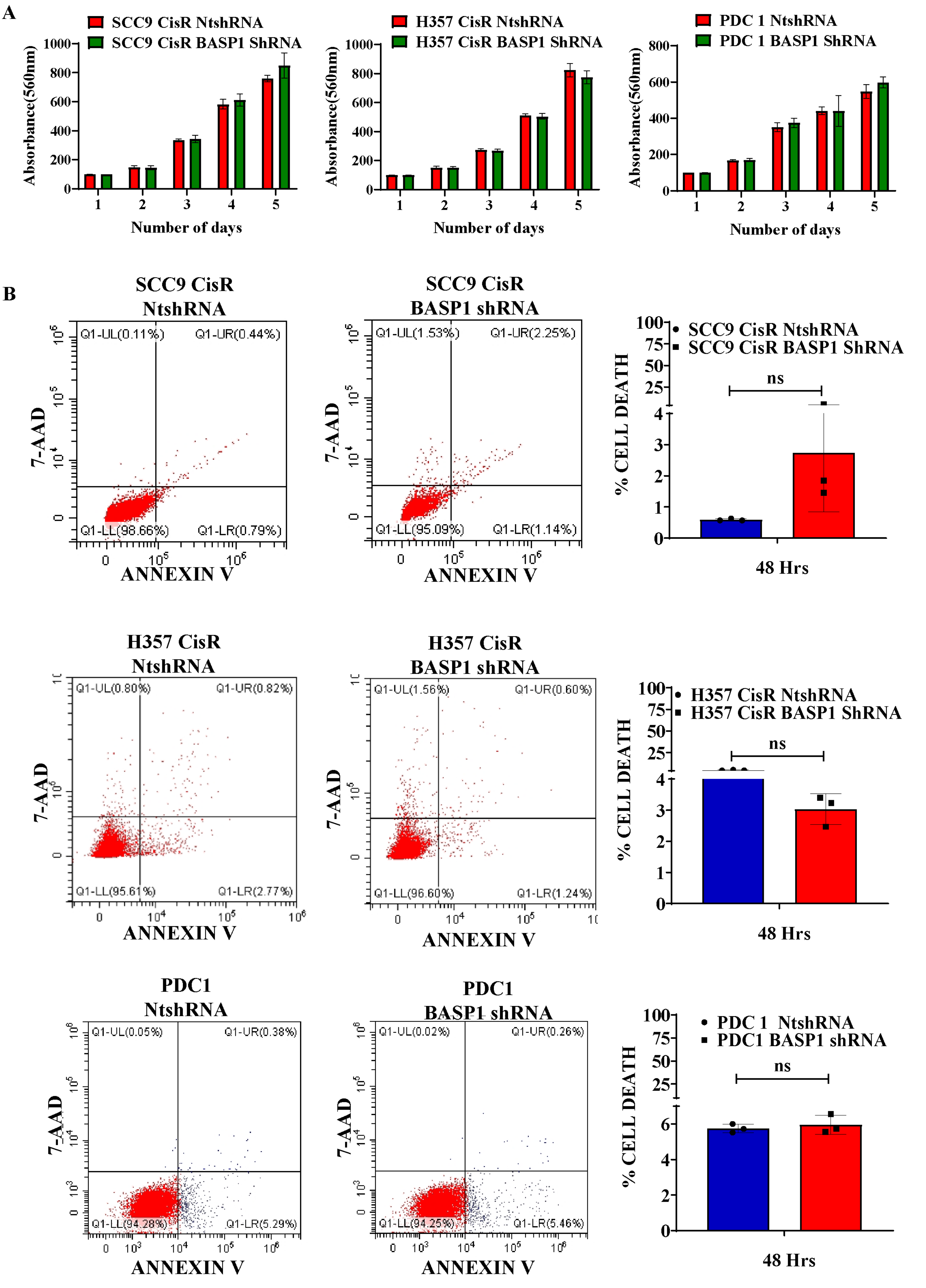

Supplementary figure 3

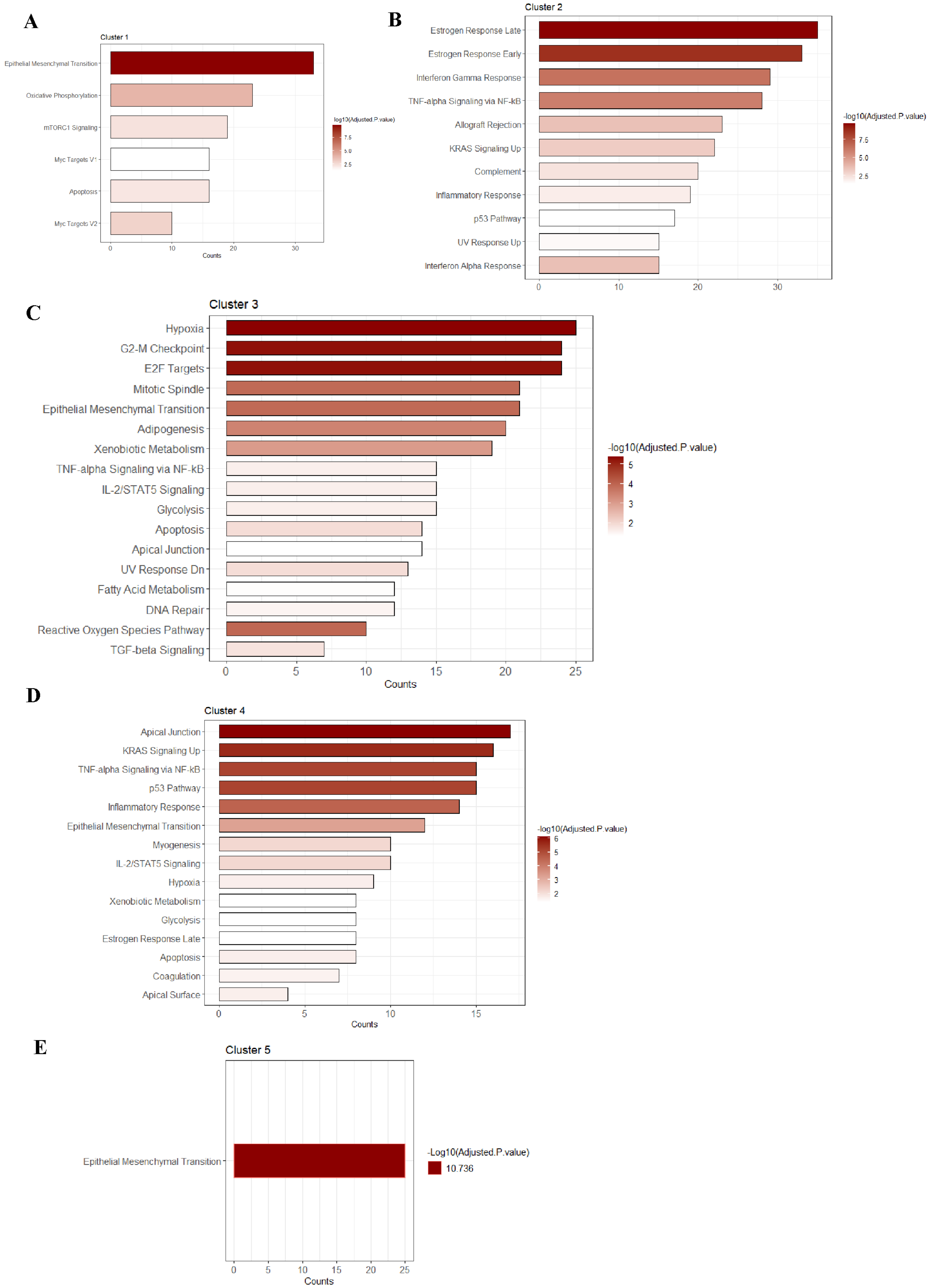

Supplementary figure 4

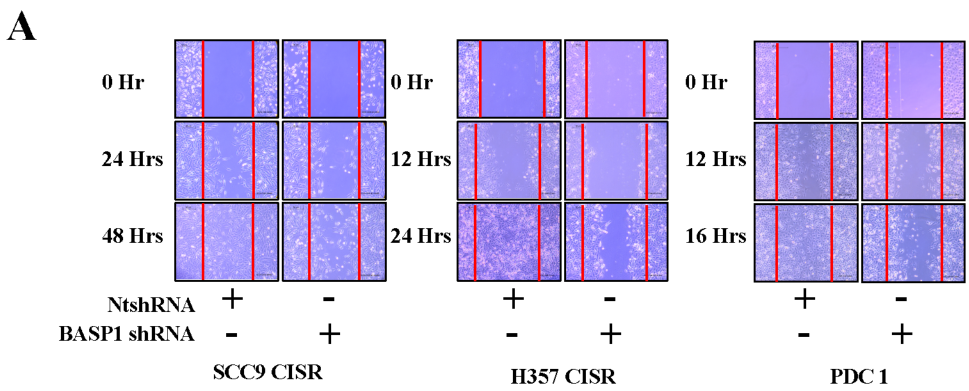

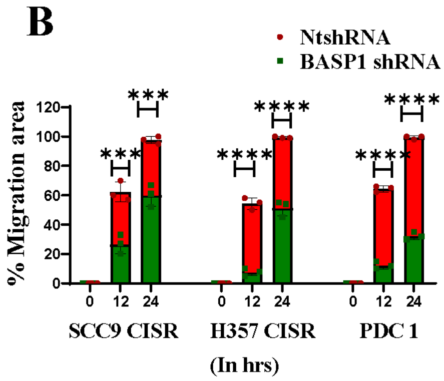

Supplementary figure 5

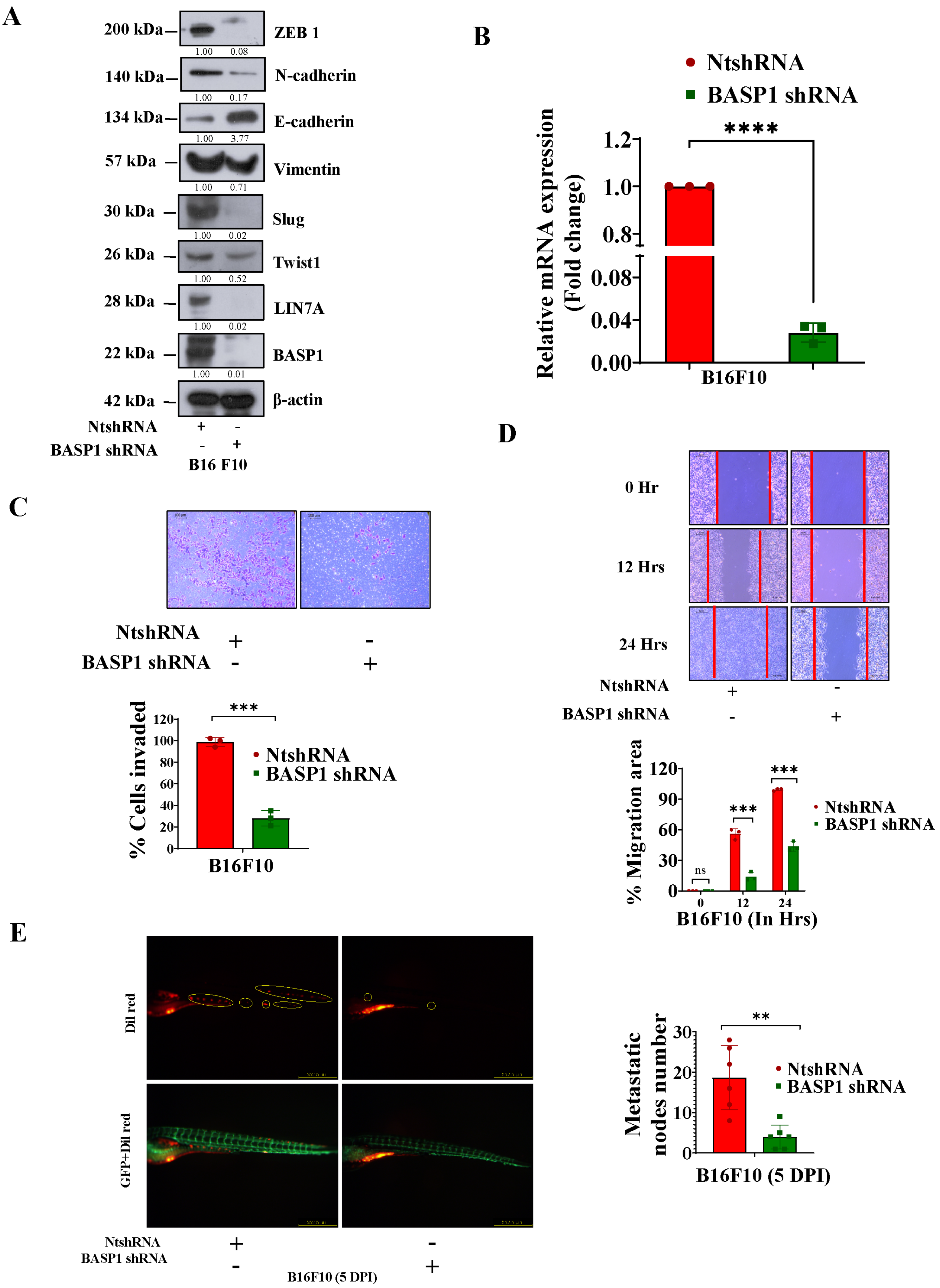

Supplementary figure 6

A

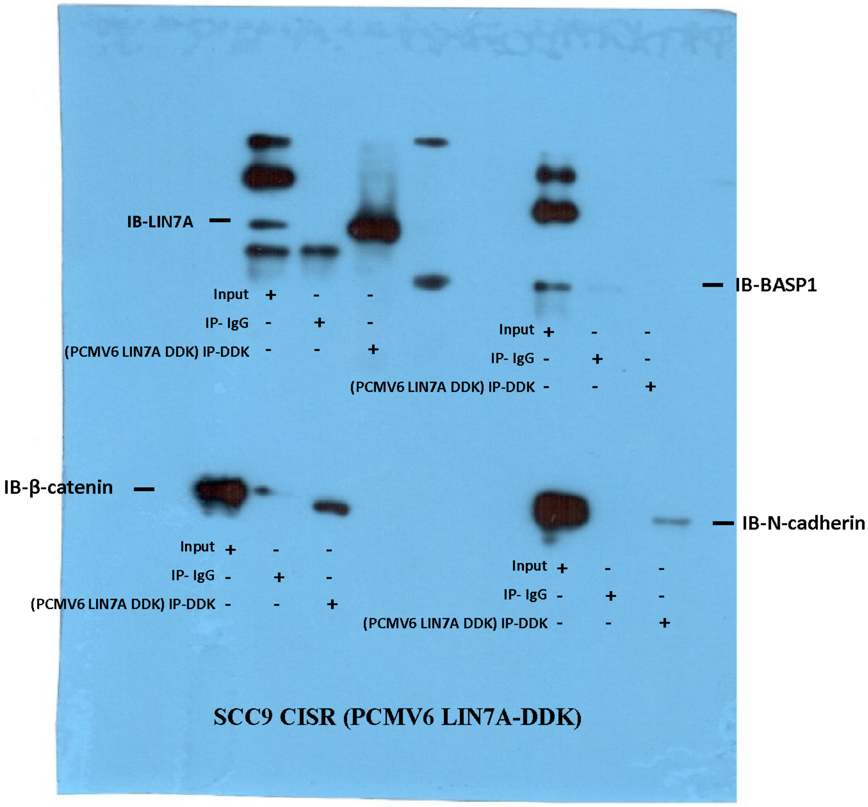

B

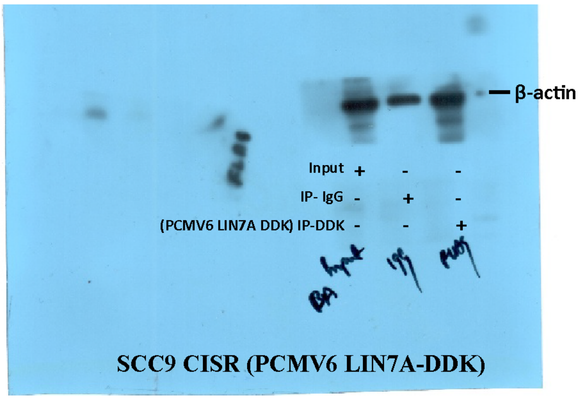

Figure S1: Single cell RNA sequencing of sensitive, early, and late chemoresistant cells:

(A) UMAP visualization of single-cell RNA-seq data, coloured by pseudotime. Cells are ordered along a developmental trajectory inferred using Slingshot. (B) Volcano plot showing the average scaled expression of BASP1 gene for annotated cell types and subclusters. (C) Summary metrics of single-cell RNA-seq data including cell counts, read depth, gene detection, and UMI counts per cell. (D) Cell distribution across clusters and subclusters in single-cell RNA-seq data, showing the number of cells per identified population.

Figure S2: Pathway enrichment analysis from different clusters: (A-E) Pathway enrichment analysis of differentially expressed genes using enrichGO in 5 clusters along the pseudotime i.e from SCC9 CisS to SCC9 8MCisR. Graph arranged on the basis of significantly deregulated pathways along their increasing gene counts.

Figure S3: BASP1 knockdown does not significantly alter proliferation and cell death: (A) Proliferation of wild type and BASP1 knockdown cells monitored by MTT assay across a 5-day time course, mean ± SEM (n=3), *$P \leq 0.05$ by 2-way ANOVA. (B) Assessment of cell viability in BASP1 knockdown cells via cell death was determined by Annexin V/7-AAD staining and flow cytometry. Dot plot with lines shows the percentage of cell death in each treatment group, mean ± SEM (n=3), *$P \leq 0.05$ by 2-way ANOVA.

Figure S4: BASP1 genomic ablation impairs tumor cell migration in chemoresistant OSCC: (A and B) Indicated NtshRNA and BASP1shRNA cells were subjected to a scratch wound healing assay as described in the Methods section. Left panel: Representative images of the scratch assay in each group. Right panel: Bar graph showing the percentage of wound closure (n=3, ***$P \leq 0.001$, ****$P \leq 0.0001$ by 1-way ANOVA).

Figure S5: BASP1 Role in EMT Regulation in a Metastatic Cell Line B16F10 (A) Cell lysates of B16F10 NtshRNA control, BASP1 shRNA from indicated cell lines were subjected to immunoblotting (n=3) using indicated antibodies. (B) Relative mRNA expression of BASP1 was analyzed by quantitative real-time PCR (qRT-PCR) in B16F10 NtshRNA control, BASP1 shRNA. Data are presented as mean ± SEM (n=3), ****$P \leq 0.0001$ by 1-way ANOVA. (C) B16F10 NtshRNA control, BASP1 shRNA cells were subjected to invasion assay as described in the Methods section. Images of the migration assay in each group are shown in a bar graph showing the relative number of migrated cells (n=3, ***$P \leq 0.001$ by 1-way ANOVA). (D) B16F10 NtshRNA control, BASP1 shRNA cells were subjected to a scratch wound healing assay as described in the Methods section. Representative images of the migration assay in each group. Bar graph showing the relative number of migrated cells (n=3, ***$P \leq 0.001$ by 1-way ANOVA). (E) Lateral view of fluorescent transgenic Tg(etsrp:EGFP) zebrafish embryos at Day 5 post-injection with Dil-Red-stained B16F10 NtshRNA control or BASP1 shRNA. Embryos metastatic nodules were assessed by counting individual nodules on Day 5. (n=6). The mean ± SD of n=6 independent experiments is shown in scatter dot plots. **$p \leq 0.01$ by 1-way ANOVA.

Figure S6: Raw blots of Co-immunoprecipitation analysis of protein LIN7A interaction with protein β-catenin, N-cadherin and BASP1 in SCC9 CisR Cell Line (A) Cell lysates of SCC9 CisR cell line was used to ectopically express LIN7A conjugated with FLAG DDK vector and subjected to immunoblotting using indicated antibodies by performing co-immunoprecipitation. (B) Cell lysates of SCC9 CisR cell line was used to ectopically express

LIN7A conjugated with FLAG DDK vector and subjected to immunoblotting using indicated antibody.

Supplementary table 01

Oligos for qRT-PCR and shRNA sequences of BASP1 and LIN7A

Table .01		
	NAME	SEQUENCE
HUMAN (Homo sapiens)	BASP1 Forward primer	CAGGAGACCAAAAGTGACGG
	BASP1 Reverse primer	TTTGGTCGGAATTAGCTGCC
	LIN7A Forward primer	GCAACAGCAAAGGCAACAGT
	LIN7A Reverse primer	CTCTTTTGAGGCCTCCGTGT
	18S Forward primer	GTAACCCGTTGAACCCCATT
	18S Reverse primer	CCATCCAATCGGTAGTAGCG
MOUSE (Mus musculus)	BASP1 Forward primer	GCGAGGCCAAAAAGACTGAG
	BASP1 Reverse primer	CCGCGCTGCTAGGTTTAGAG
	18S Forward primer	GTAACCCGTTGAACCCCATT
	18S Reverse primer	CCATCCAATCGGTAGTAGCG
shRNA	BASP1 3'UTR shRNA Forward	CCGGGCACCTGTAGTTCTGTTTATTCTCGAGAATAAACAGAACTACAGGTGCTTTTTG
	BASP1 3'UTR shRNA Reverse	AATTCAAAAAGCACCTGTAGTTCTGTTTATTCTCGAGAATAAACAGAACTACAGGTGC
	LIN7A 3'UTR shRNA Forward	CCGGGCTTCAGAATCCCAGCACATACTCGAGTATGTGCTGGGATTCTGAAGCTTTTTG
	LIN7A 3'UTR shRNA Reverse	AATTCAAAGCTTCAGAATCCCAGCACATACTCGAGTATGTGCTGGGATTCTGAAGC
	NTshRNA Forward	CCGGCAACAAGATGAAGAGCACCAACTCGAGTTGGTGCTCTTCATCTTGTTGTTTTTG
	NTshRNA Reverse	AATTCAAAAACAACAAGATGAAGAGCACCAACTCGAGTTGGTGCTCTTCATCTTGTTG
miRNA	U6 forward primer	GAGGGCCTATTTCCCATGATT
	miR-501-3p Forward primer	AATGCACCCGGGCAAGGATTCT

Supplementary table 02 A

OSCC patient details of lymph node positive tissue IHC				
Sl No	Tumor samples	Age/Sex	Site Of Disease	Clinical stage
1	Patient 01	32/M	Tongue lateral border	T4N1MO
2	Patient 02	42/M	Right tongue	T4N1M0
3	Patient 03	60/M	Left buccal mucosa	T4aN1M0
4	Patient 04	33/M	Left lateral tongue border	T3N1MX
5	Patient 05	55/F	Right buccal mucosa	T4BN2bMO
6	Patient 06	50/M	Right buccal mucosa	T4aN2M0
7	Patient 07	45/M	Oral cavity	T2N1M0
8	Patient 08	45/M	Oral cavity	T4N1M0
9	Patient 09	75/M	Oral cavity	T4AN2bM0
10	Patient 10	55/M	Tongue	T4aN2cM0
11	Patient 11	48/M	SCC base of tongue with extension into oropharynx	T4aN2cMO
12	Patient 12	38/M	Right buccal mucosa	T4bN2bMO
13	Patient 13	34/M	Left buccal mucosa	T4aN2bMx
14	Patient 14	38/M	Right gingiva buccal sulcus	T4N1Mx
15	Patient 15	40/M	Tongue	T2N2cM0
16	Patient 16	45/M	Tongue, well differentiated	T2N1M0
17	Patient 17	M/32	Right lateral border of tongue	T4N1M0
18	Patient 18	29/F	Right upper maxillary	T4N2M0
19	Patient 19	60/F	Upper alveolus	T4aN2M0
20	Patient 20	60/M	Left buccal mucosa	T4N3M0
21	Patient 21	42/M	Tongue	T4N2M0
22	Patient 22	40/F	Carcinoma sulcus (LEFT)	T3Mx
23	Patient 23	52/M	Carcinoma buccal mucosa	T2Nx
24	Patient 24	30/M	Carcinoma -bm (LEFT)	T2N1
25	Patient 25	45/M	Carcinoma - tongue (RIGHT)	T2N2b
26	Patient 26	45/F	Lesion over left side tongue	T2N2b
27	Patient 27	60/M	Carcinoma palate (Right)	T3N3bMx
28	Patient 28	52/M	Carcinoma bm (Right)	T3N1Mx.
29	Patient 29	45/M	Carcinoma tongue	T3N1
30	Patient 30	52/M	Carcinoma bm (Right)	T3N1Mx.
31	Patient 31	41/F	Right cheek	T4aN2b
32	Patient 32	33/M	Carcinoma (Left)	T4aN2b
33	Patient 33	41/F	Right cheek	T4aN2b
34	Patient 34	38/F	Buccal mucosa (Right)	T4a N2b
35	Patient 35	63/F	Lower gum (Right)	T4aN1 M0
36	Patient 36	51/M	Left buccal	T4aN3bMx.
37	Patient 37	55/M	Carcinoma - (left) desmoplasia	T4aNx
38	Patient 38	32/M	Carcinoma lower alveolic buccal mucosa	T4aN1M0
39	Patient 39	33/M	Carcinoma left	T4aN1M0
40	Patient 40	64/F	Carcinoma lower adenocarcinoma	T4aN3bM0

41	Patient 41	59/F	Right tongue	T4aN2cM0
42	Patient 42	44/M	Buccal mucosa	T4aN2aM0
43	Patient 43	52/M	Buccal mucosa	T2N1M0
44	Patient 44	36/M	Buccal mucosa	T4aN2M0
45	Patient 45	42/F	Right buccal	T4aN3bM0
46	Patient 46	36/M	Lateral tongue	T4aN3bM0
47	Patient 47	53/M	Right lower mandibular	T4aN1M0
48	Patient 48	41/M	Left upper lip and mucosa	T2N1M0
49	Patient 49	48/M	Right lower gingiva	T2N2MX
50	Patient 50	58/F	Left gingiva buccal	T4AN1Mx
51	Patient 51	68/M	Left buccal mucosa	T4aN1MO
52	Patient 52	34/F	Left buccal mucosa	T4N2bM0
53	Patient 53	61/M	Central; arch mouth	T4aN2M0
54	Patient 54	41/M	Lower border tongue	T2N1M0
55	Patient 55	70/M	Right buccal mucosa	T2N2bM0

Supplementary table 02 B

OSCC patient details of lymph node negative tissue IHC				
Sl No	Tumor samples	Age/Sex	Site Of Disease	Clinical stage
1	PATIENT 56	74/M	Tongue	T2N0M0
2	PATIENT 57	53/M	Tongue	T4aN0M0
3	PATIENT 58	45/M	Right mandible	T4bN0Mx
4	PATIENT 59	M/55	Upper lip	T4N0M0
5	PATIENT 60	M/40	Lateral border of tongue, mod. Differenciated	T4aN0M0
6	PATIENT 61	54/M	Carcinoma left buccal mucosa	T1N0
7	PATIENT 62	65/M	Buccal mucosa (Left)	T1N0
8	PATIENT 63	70/F	Carcinoma tongue (Left)	T2N0
9	PATIENT 64	33/M	Buccal mucosa (Left)	T2N0
10	PATIENT 65	45/M	Carcinoma bm (Left), wound in Left cheeck from 1 month	T2N0
11	PATIENT 66	40/M	Carcinoma tongue	T2N0
12	PATIENT 67	70/F	Carcinoma tongue (Left)	T2N0
13	PATIENT 68	28/M	Carcinoma -bm (Left)	T2N0
14	PATIENT 69	71/M	Case of lesion left side oral cavity	T2N0
15	PATIENT 70	70/M	Carcinoma tongue (Left)	T3N0
16	PATIENT 71	56/M	Carcinoma angle of mouth (Left)	T3N0M0
17	PATIENT 72	72/M	Carcinoma oral cavity taken (advanced)	T4N0
18	PATIENT 73	65/M	Left side oral cavity	T4aN0
19	PATIENT 74	66/M	Carcinoma tongue, large growth (Left)	T4N0Mx
20	PATIENT 75	45/M	Left buccal	T4aN0
21	PATIENT 76	42/M	Buccal mucosa	T4aN0M0
22	PATIENT 77	52/M	Right buccal mucosa	T3N0M0
23	PATIENT 78	59/M	Right buccal mucosa	T4aN0M0
24	PATIENT 79	65/F	Left buccal mucosa	T1N0M0
25	PATIENT 80	44/M	Carcinoma right tongue	T4aN0M0
26	PATIENT 81	34/M	SCC right buccal mucosa	T2N0M0
27	PATIENT 82	70/M	Upper alveolus buccal mucosa	T4aN0M0
28	PATIENT 83	55/M	Lower lip	T4aN0M0
29	PATIENT 84	35/M	Carcinoma buccal	T4aN0M0
30	PATIENT 85	49/M	Buccal mucosa	T2N0M0
31	PATIENT 86	30/M	Right tongue	T3N0M0
32	PATIENT 87	39/M	Right lower gingivocal	T2N0
33	PATIENT 88	36/M	Right lateral border of tongue	T2N0M0
34	PATIENT 89	35/M	Buccal mucosa and lower lip	T4aN0M0
